# *Plasmodium falciparum* Blood Stage Antimalarial Vaccines: An Analysis of Ongoing Clinical Trials and New Perspectives Related to Synthetic Vaccines

**DOI:** 10.3389/fmicb.2019.02712

**Published:** 2019-12-03

**Authors:** David Ricardo Salamanca, Marcela Gómez, Anny Camargo, Laura Cuy-Chaparro, Jessica Molina-Franky, César Reyes, Manuel Alfonso Patarroyo, Manuel Elkin Patarroyo

**Affiliations:** ^1^Fundación Instituto de Inmunología de Colombia, Bogotá, Colombia; ^2^Ph.D. Programme in Biomedical and Biological Sciences, Universidad del Rosario, Bogotá, Colombia; ^3^Medicine Programme, Health Sciences Faculty, Universidad de Boyacá, Tunja, Colombia; ^4^Basic Sciences Department, School of Medicine and Health Sciences, Universidad del Rosario, Bogotá, Colombia; ^5^Department of Pathology, School of Medicine, Universidad Nacional de Colombia, Boyacá, Colombia

**Keywords:** clinical trial, immunogenicity, vaccine, malaria, antimalarial vaccine, merozoite, *Plasmodium falciparum*

## Abstract

*Plasmodium falciparum* malaria is a disease causing high morbidity and mortality rates worldwide, mainly in sub-Saharan Africa. Candidates have been identified for vaccines targeting the parasite’s blood stage; this stage is important in the development of symptoms and clinical complications. However, no vaccine that can directly affect morbidity and mortality rates is currently available. This review analyzes the formulation, methodological design, and results of active clinical trials for merozoite-stage vaccines, regarding their safety profile, immunological response (phase Ia/Ib), and protective efficacy levels (phase II). Most vaccine candidates are in phase I trials and have had an acceptable safety profile. GMZ2 has made the greatest progress in clinical trials; its efficacy has been 14% in children aged less than 5 years in a phase IIb trial. Most of the available candidates that have shown strong immunogenicity and that have been tested for their protective efficacy have provided good results when challenged with a homologous parasite strain; however, their efficacy has dropped when they have been exposed to a heterologous strain. In view of these vaccines’ unpromising results, an alternative approach for selecting new candidates is needed; such line of work should be focused on how to increase an immune response induced against the highly conserved (i.e., common to all strains), functionally relevant, protein regions that the parasite uses to invade target cells. Despite binding regions tending to be conserved, they are usually poorly antigenic and/or immunogenic, being frequently discarded as vaccine candidates when the conventional immunological approach is followed. The Fundación Instituto de Inmunología de Colombia (FIDIC) has developed a logical and rational methodology based on including conserved high-activity binding peptides (cHABPs) from the main *P. falciparum* biologically functional proteins involved in red blood cell (RBC) invasion. Once appropriately modified (mHABPs), these minimal, subunit-based, chemically synthesized peptides can be used in a system covering the human immune system’s main genetic variables (the human leukocyte antigen HLA-DR isotype) inducing a suitable, immunogenic, and protective immune response in most of the world’s populations.

## Introduction

*Plasmodium falciparum* malaria continues being one of the main diseases having a great impact on public health worldwide. It has been estimated that 219 million clinical cases and more than 435,000 malaria-related deaths occurred worldwide in 2017, most of them (90%) in sub-Saharan Africa (WHO, 2018). In spite of many efforts at early diagnosis, timely treatment, and attempts at producing antimalarial vaccines, a completely effective strategy for controlling malaria has not yet been achieved.

**FIGURE 1:**
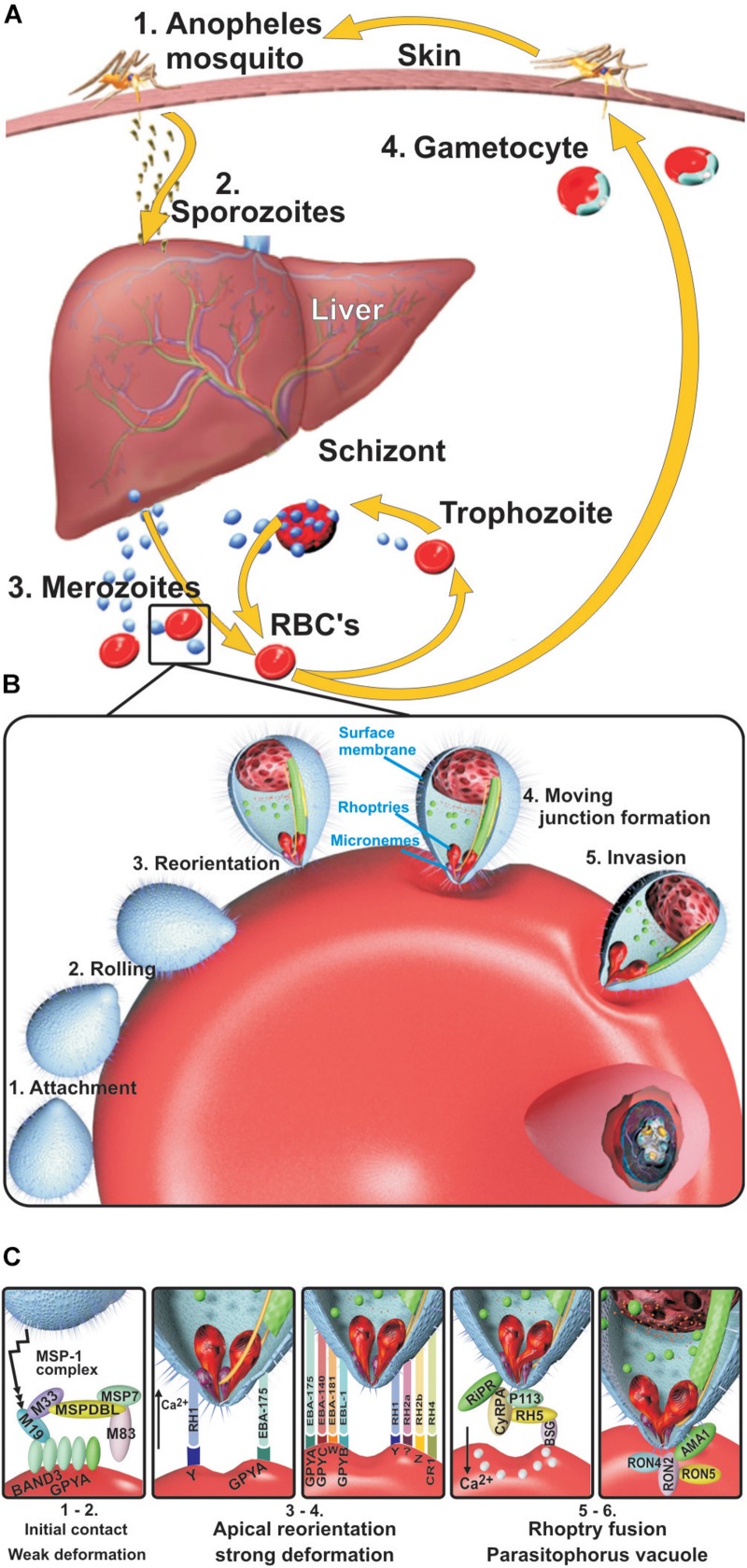
*Plasmodium falciparum* life cycle. **(A)** Liver stage. Sporozoites (Spz) inoculated during the bite of a female *Anopheles* mosquito migrate to hepatic cells through the host’s blood stream, thereby infecting hepatocytes where they reproduce (30,000–50,000 per Spz) (pre-erythrocyte cycle) and become transformed into merozoites (Mrz). When Mrz are released into the blood stream, they invade the red blood cells (RBCs) (erythrocyte cycle), producing 30–50 new Mrz every 48 h during which the cycle lasts, inducing the release of subproducts, immune system molecule production, and the symptoms of the disease, which can cause death in some people. Some Mrz become gametocytes, which then become digested by other mosquitos during fresh biting to start their sexual cycle and produce new Spz. **(B)** Blood stage. Mrz roll over RBC and adhere to their surface. They become reorientated toward their apical pole by high-affinity interactions between microneme proteins and erythrocyte receptors, thereby deforming the RBC membrane to create a tight junction (TJ) to enable their entry. The rhoptry proteins are then released onto the RBC surface, and the parasitophorous vacuole membrane (PVM) starts forming; the parasite progressively develops after entry into ring, trophozoite, and schizont forms. **(C)** Mrz interaction with ligands and their erythrocyte receptors. The MSP1 complex interacts with BAND3-GPYA, EBA-140 with GYPC, EBA-175 with GYPA, EBA-181 with the E receptor, EBL1 with GYPB, RH1 with the Y receptor, RH2b with the Z receptor, RH4 with CR1, and RH5 with BSG. GYPA: glycophorin A, GYPB: glycophorin B, GYPC: glycophorin C, BSG: basigin, PVM: parasitophorous vacuole membrane, CR1: complement receptor 1.

There are several ongoing approaches to developing vaccines targeting the different stages of the *P. falciparum* parasite’s life cycle (Figure 1A), including the asexual phase in human hepatic and erythrocyte cells and the sexual phase in the female *Anopheles* mosquito (Koram and Gyan, 2010; Coelho et al., 2017).

Understanding the intra-erythrocyte cycle which includes red blood cell (RBC) invasion arouses major interest since the clinical symptoms of malaria are manifest during this stage, characterized by very high fever, headache, nausea, vomiting, and general malaise (Fletcher and Beeching, 2013; Tahita et al., 2013). Multiple hematological complications also occur, such as anemia, hemolysis, and hemagglutination, during the parasite’s cyclic invasion, growth, and reproduction (Ord et al., 2015), which can lead to renal and cerebral damage, vasculitis, and patients’ rapid death.

Erythrocyte invasion (Figure 1B) begins when merozoites (Mrz) roll on the RBC surface via receptor–ligand low-affinity interactions, mainly carried out by some of the 12-Mrz surface protein (MSP) family (Rodríguez et al., 2005) (MSP1 to MSP12), inducing weak erythrocyte membrane deformation (Weiss et al., 2015). The Mrz then become reorientated, inducing strong calcium (Ca^++^)-mediated, actin-dependent RBC deformation via the erythrocyte binding antigen (EBA-175, EBA-140, and EBA-181) and erythrocyte binding ligand (EBL) and reticulocyte binding homolog (Rh1, Rh2a/Rh2b, and Rh4) families. The latter acts as an alternative pathway to EBA, depending on the presence or absence of RBC membrane receptor molecules to bring their apical poles into direct contact with the host cell membrane. This induces tight junction (TJ) formation between RBC proteins and Mrz receptor molecules immediately after the Rh5–basigin interaction has induced the rhoptries to release microneme proteins, leading to rhoptry neck (RON-2) (Cao et al., 2009) and apical membrane antigen-1 (AMA-1) complex formation interacting with a yet-unknown receptor (Weiss et al., 2015) (Figure 1C).

Mrz invasion continues via the interaction of rhoptry proteins, dense granules, and RBC membrane lipids participating in the formation of the parasitophorous vacuole membrane (PVM). This enables parasite multiplication (32–50 per cycle/48–72 h) through the sequential formation of ring, trophozoite, and schizont stages (Cowman et al., 2012, 2016) (Figure 1C).

A vaccine targeting the erythrocyte stage capable of interrupting *P. falciparum*’s asexual cycle could reduce parasitemia and morbidity by blocking invasion and the production of harmful by-products during parasite growth, which induces clinical symptomatology (Miura, 2016; Patarroyo et al., 2017a; Draper et al., 2018). Several blood-stage antigens have thus been identified as potential vaccine candidates; this led to FIDIC developing the first minimum subunit-based synthetic multi-epitope, multistage vaccine 30 years ago: synthetic *P. falciparum* vaccine 66 (SPf66). That vaccine contained three Mrz-derived protein fragments [MSP1, SERA, and an amino acid (aa) sequence from a then-undefined molecule] and the Spz CSP1-derived asparagine–alanine–asparagine–proline (NANP) sequence, interspersed twice. It had ∼45% protective efficacy in the *Aotus* monkey experimental model and induced 40% full protection in a controlled human malaria infection (CHMI) trial and in large phase III field trials involving a population aged over 1 year in Colombia, Venezuela, Ecuador, and Brazil (∼50,000 people vaccinated) (Patarroyo, 1987, 1988; Valero et al., 1993). An SPf66 vaccine produced in the United States by another group, having a different degree of polymerization, induced limited protection which was not statistically significant in a field trial in Thailand (Nosten et al., 1996). These results clearly suggested that SPf66 vaccine’s protective efficacy was genetically (later confirmed by HLA-DR phenotyping) and transmission intensity dependent and SPf66 vaccination was thus stopped to continue with the search for more epitopes that could form part of a fully protective vaccine.

The WHO’s latest update (WHO, 2017) states that many vaccines (>100) have been tested on humans since SPf66. Only the following 10 recombinant or viral vaccine candidates are currently in clinical studies: EBA region II-non-glycosylated (EBA-175 RII NG), apical membrane antigen 1 diversity covering (AMA-1 DiCo), Mrz surface protein-3 (MSP3) long synthetic peptide (LSP), serine repeat antigen-5 formulated with aluminum hydroxyl gel (BK-SE36), *P. falciparum* MSP3 and glutamate-rich protein (GLURP) (GMZ2) fusion protein, the chimpanzee adenovirus 63 (ChAd63) with modified vaccinia virus Ankara (MVA) encoding the reticulocyte binding homolog-5 (ChAd63/MVA RH5), two placental malaria vaccine candidates (PRIMVAC and PAMVAC), unstructured 104mer synthetic peptide (P27A), and pre-erythrocytic and blood-stage (PEBS) vaccines (Table 1).

**TABLE 1:** Current clinical trials for *Plasmodium falciparum* erythrocyte stage vaccine candidates.

**Vaccine formulation**	**Trial characteristics**		**Main results**	**References**
**Chemically synthesized vaccine** **MSP3-LSP** **Adjuvants:** Montanide ISA 720 and aluminum hydroxide	**Phase Ia**	**Doses evaluated:** 10, 30, 100, and 300 μg of MSP3-LSP (days 0, 30, and 120) **Administration route:** Subcutaneous **Participants:** 36 (18–45 years old) **Year:** 2003–2004	– formulation with Al(OH)_3_ was better tolerated – cytophilic IgG1 subclass humoral response predominated	Audran et al., 2005; Sirima et al., 2009
	**Phase Ib**	**Dose:** 30 μg of MSP3-LSP (days 0, 28, and 112) **Participants:** Group 1: 30 (18–45 years old); Group 2: 55 (12–24 months old) **Year:** 2007–2008	– vaccine was safe and well tolerated – absence of immune humoral response in the vaccinated group	
**Chemically synthesized vaccine** **P27A** **Adjuvants:** GLA-SE/alhydrogel	**Phase Ia**	**Dose:** 10 and 50 μg of P27A (days 0, 28, and 56) **Administration route:** Intramuscular **Participants:** 16 adults **Year:** 2014–2015	– high frequency of local and systemic adverse events (AE)	Steiner-Monard et al., 2018
	**Phase Ib**	**Dose:** 10 and 50 μg of P27A (days 0, 28, and 56) **Administration route:** Intramuscular **Participants:** 40 adults **Year:** 2014–2015	– > humoral immune response in individuals who had not been exposed to malaria – low antibody dependent (ADCI) – anti-P27A cell inhibition capability (*in vitro* trials)	
**Recombinant vaccine** **BK-SE36** **Adjuvant:** aluminum hydroxide **Expression system (ES):** *Escherichia coli*	**Phase Ia**	**Dose evaluated:** 50 and 100 μg of SE36 (days 0, 21, and 42) **Administration route:** Subcutaneous **Participants:** 40 adults **Year:** 2008	– 100% seroconversion (29/29 participants) at the end of follow-up (day 63)	Horii et al., 2010; Palacpac et al., 2013
	**Phase Ib**	**Dose:** 50 and 100 μg of SE36 (days 0 and 21) **Participants:** Stage 1: 56 (21–40 years old)/Stage 2: 84 (6–20 months old) **Year:** 2010–2011	– significant increase in Ab titers only in 6- to 10-year-old participants (5/71-fold)	
**Recombinant vaccine** **AMA-1 DiCo** **Adjuvants:** aluminum hydroxide, GLA-SE **ES:** *Pichia pastoris*	**Phase Ia**	**Dose evaluated:** 50 μg of AMA-1 DiCo (days 0, 28, and 182) **Administration route:** Intramuscular **Participants:** 30 (20–25 years old) **Year:** 2014–2015	– average IgG concentration: adjuvant GLA-SE (37.7–60.2 mg/ml) – alhydrogel (19–22.9 mg/ml)	Sirima et al., 2017
	**Phase Ib**	**Dose evaluated:** 50 μg of AMA-1 DiCo (days 0, 28, and 182) **Participants:** 36 (20–25 years old) **Year:** 2014–2015	– significant increase in IgG (90–150 mg/ml) during week 30 in the GLA-SE group	
**Recombinant vaccine** **PRIMVAC** **Adjuvants:** alhydrogel and GLA-SE **ES:** *E. coli*	**Phase Ia/Ib**	**Dose evaluated:** 20, 50, and 100 μg of PRIMVAC (days 0, 28, and 56) **Administration route:** Intramuscular **Participants:** 68 (18–35 years old) **Year:** 2016–2018	– not published	(Chêne et al., 2016; Sirima et al., 2016a)
**Recombinant vaccine** **PAMVAC** **Adjuvants:** alhydrogel and GLA-SE **ES:** *Drosophila melanogaster*	**Phase Ia/Ib**	**Dose evaluated:** 20, 50, and 100 μg of PRIMVAC (days 0, 28, and 56) **Administration route:** Intramuscular **Participants:** 66 (18–35 years old) **Year:** 2016–2017	– not published	(Chêne et al., 2016)
**Recombinant vaccine** **PfPEBS** **Adjuvants:** aluminum hydroxide **ES:** *E. coli*	**Phase Ia/Ib**	**Dose evaluated:** 5 or 30 μg of PfPEBS (days 0 and 28) **Administration route:** Subcutaneous **Participants:** 36 (18–45 years old)	– not published	Druilhe and Genton, 2012
**Recombinant vaccine** **EBA-175 RII NG** **Adjuvant:** aluminum phosphate **ES:** *P. pastoris*	**Phase Ia**	**Dose:** 5, 20, 80, and 160 μg of EBA-175 (days 0, 42, and 194) **Administration route:** Intramuscular **Participants:** 80 adults **Year:** 2008	– vaccine well tolerated – > Ab titers following third immunization; however, becoming reduced at the end of follow-up	El Sahly et al., 2010
	**Phase Ib**	**Dose:** 5, 20, and 80 μg of EBA-175 (days 0, 42, and 194) **Administration route:** Intramuscular **Participants:** 60 adults **Year:** 2010–2012	– inhibition of parasite growth *in vitro*: 25.0% in the placebo group and 12.0–20.0% in the vaccinated group	Koram et al., 2016
**Recombinant vaccine** **GMZ2** **Adjuvant:** alhydrogel **Expression system:** *Lactococcus lactis*	**Phase Ia**	**Dose:** 10, 30, or 100 μg of GMZ2 (days 0, 28, and 56) **Administration route:** subcutaneous **Participants:** 30 adults **Year:** 2006–2007	– high frequency of local and systemic adverse reactions (grades 1 and 2)	Esen et al., 2009
	**Phase Ib**	**Dose:** 100 μg of GMZ2 (days 0, 28, and 56) **Participants:** 40 adults (18–45 years old) **Year:** 2007–2008	– greater amount of anti-GMZ2 Ab titers in the experimental group (>1.4-fold)	Mordmüller et al., 2010
	**Phase IIb**	**Dose:** 100 μg of GMZ2 (days 0, 28, and 56, 1-year follow-up) **Participants:** 1,849 children (1 and 5 years old) **Year:** 2010–2014	– efficacy: 14%	Bélard et al., 2011; Sirima et al., 2016b
**Recombinant adenovirus vaccine** **ChAd63 RH5/MVA RH5** **ES adenoviral:** ChAd63 and MVA	***Phase Ia***	**Dose:** – ChAd63 RH5 [5 × 10^9^ viral particles (vp) cf. 5 × 10^10^ vp] – ChAd63 [5 × 10^10^ vp + MVA RH5 1 × 10^8^ plaque-forming unit (pfu)]/(5 × 10^10^ vp + MVA RH5 2 × 10^8^ pfu) **Participants:** 24 non-exposed adults (19–49 years old) **Year:** 2014–2015	– suitable safety profile – inhibition of parasite growth *in vitro* (GIA): 36.0 and 50.6%	Payne et al., 2017

This review covers the results of current clinical trials carried out with these vaccine candidates, analyzing the formulation, aa sequence, and 3D structure of some fragments of these proteins selected for developing vaccine candidates and their relationship with conserved high activity binding peptides (cHABPs) identified in our institution. A large amount of our cHABPs’ aa sequences (besides mediating RBC binding and invasion) have been found in vaccine candidate fragments as they are located in such proteins’ functionally strategic sites (i.e., nutrient and calcium ion transport and enzymatic activities). These cHABPs thus represent excellent targets for developing synthetic vaccines since they can block the binding of proteins involved in RBC invasion as well as some of their biological functions.

The methodological design of current clinical trials and analysis of their results are reviewed, discussing reported safety, immunogenicity, and efficacy data.

## Clinical Studies Regarding Blood-Stage Malaria Vaccines

### MSP3-LSP Vaccine

The *P. falciparum* MSP3, secreted and translocated to a parasite membrane, has ∼43-kDa molecular weight (Deshmukh et al., 2018). An LSP has been chemically synthesized from the 95-aa- long MSP3 fragment in its C-terminal extreme’s conserved region, including the (^181^RKTKEYAEKAKNAYEKAKNA**YQKANQAVLKAKEASS**YDYILGWEFGGGVPEHKKEENMLSHLYVSSKDKENISKENDDVLDEKEEEAEETEEEELE^276^) aa sequence (Audran et al., 2005; Sirima et al., 2007).

MSP3-LSP vaccine candidate results have been published in phase Ia/Ib clinical trial (Sirima et al., 2007, 2009; Nebie et al., 2009). Phase Ia carried out in Switzerland involved 35 healthy volunteers; three subcutaneous injections of MSP3-LSP were administered in 10-, 30-, 100-, and 300-μg doses on days 0, 30, and 120. Montanide ISA 720 and aluminum hydroxide (AH) adjuvants were used, AH being better tolerated and having a lower reactogenicity rate. It did not lead to serious vaccine-related adverse events (AE) during the trial. Regarding immunogenicity results, 63% (22/35) of the volunteers had detectable antibody (Ab) levels against MSP3-LSP following the second dose and 77% (23/30) following the third dose (ranging from 200 to 3,200 μg/ml, average 1,600 μg/ml); however, these titers became dramatically reduced by the end of follow-up (month 12) (Audran et al., 2005).

MSP3-LSP was considered safe in phase Ib trials in Burkina Faso involving thirty 18- to 45-year-old healthy volunteers who were vaccinated with three doses of 30 μg on days 0, 28, and 112; there was only one systemic AE (tachycardia) which became spontaneously resolved, with no significant laboratory alterations being observed. There was a total absence of humoral response in all subjects vaccinated with MSP3-LSP, which could have been associated with preexisting levels of humoral immunity due to previous exposure to *P. falciparum.* It was thus suggested that a clinical trial should be performed using small children who had not acquired immunity (Sirima et al., 2007).

A stepped-dose trial was subsequently carried out involving 45 children between 12 and 24 months old who were randomized for receiving a 15- or 30-μg dose of MSP3-LSP. The vaccine was well tolerated regarding both doses, and no severe AE were observed. There was greater IgG1 and IgG3 subclass cytophilic Ab production, having a better response in participants vaccinated with the 30-μg dose. MSP3-LSP was considered to induce a suitable humoral immune response in less than 2-year-old children and was safe; it was decided that it should be evaluated in biological *in vivo* trials involving a wider population and including malaria challenge trials (Sirima et al., 2009). Phase II trial results remain unknown, as no reproducible and/or positive results have been obtained regarding the study population.

### P27A Vaccine

**FIGURE 2:**
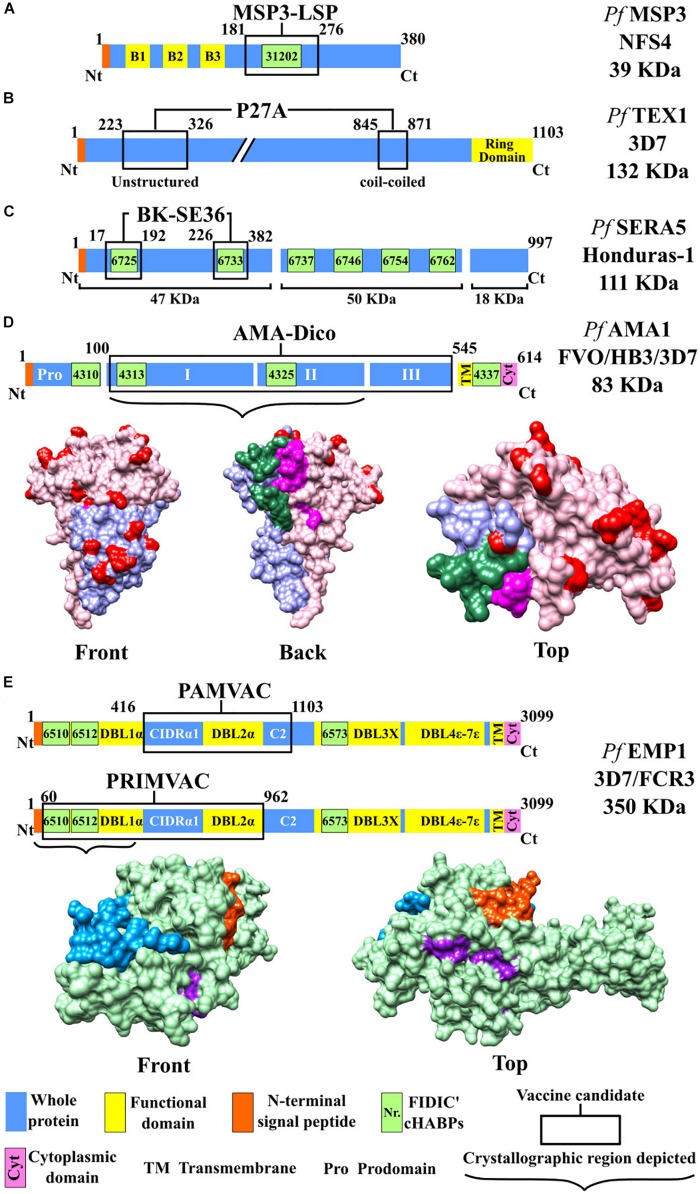
Scheme for proteins used as candidates for an antimalarial vaccine (I). Color code: blue, whole protein; yellow, functional domain; orange, N-terminal signal peptide; green, conserved high-activity binding peptides (cHABPs) with Fundación Instituto de Inmunología de Colombia (FIDIC)-assigned coding; purple, cytoplasmic domain. **(A)** MSP3-LSP vaccine, including B1, B2, and B3 domains (yellow). **(B)** P27A vaccine, including ring domain (yellow). **(C)** BK-SE36 vaccine. **(D)** AMA-1-DiCo vaccine, including domain I (purple), domain II (red), variable amino acids (pink), and cHABPs 4325 (green) and 4313 (magenta). PDB 4R19 (Lim et al., 2014). **(E)** PAMVAC and PRIMVAC vaccines. Top: Protein scheme, CIDRα1 (cysteine-rich inter-domain regions α1), C2 (C2 domain), and Duffy binding-like (DBL) domain. Bottom: Protein structure, including the NTS region (blue), whole protein (green), and cHABPs 6510 (purple) and 6512 (orange). PDB 2XU0.

P27A is an unstructured 104mer synthetic peptide from the *P. falciparum* TEX1 blood-stage protein. Developing this vaccine involved making bioinformatic predictions for protein structures including α-helix coil-type motifs; they were based on the hypothesis that the best vaccine candidates have this type of secondary structure and that they can imitate native *P. falciparum* epitopes in an aqueous environment, thereby facilitating immune system recognition (Corradin et al., 2007). Such analysis led to identifying the trophozoite export protein (Tex1) encoded by the *PFF0165c* gene which was chemically synthesized in short peptides, the P27 α-helix region (^845^K–^871^T), and the P27A intrinsically unstructured N-terminal region (^223^H–^326^S) (Figure 2B) which is characterized by having few hydrophobic aa and high hydrophilic content (Villard et al., 2007).

*In vitro* analysis of the Tex1 protein indicated that it had low polymorphism and was exported by the PVM (Kulangara et al., 2012). Preclinical studies demonstrated that polypeptide P27A produced high immunogenicity when formulated with different adjuvants; it was well recognized in mouse and rabbit animal models (Remarque et al., 2008a), thereby agreeing with the results from *in vitro* analysis of sera and peripheral blood from semi-immune adults naturally exposed to malaria (Olugbile et al., 2009).

A phase Ia/Ib clinical study was sequentially carried out on 16 Swiss and 40 Tanzanian volunteers who were given three doses of 10 and 50 μg of P27A on days 0, 28, and 56, with glucopyranosyl lipid adjuvant-stable emulsion (GLA-SE) and alhydrogel adjuvants. The vaccine’s safety and tolerability were evaluated during an 8-month follow-up; slight and moderated AE were reported in 100% of the volunteers involved in phase Ia and in 82.5% of those in phase Ib, having a higher reactogenicity rate with the GLA-SE adjuvant (Steiner-Monard et al., 2018).

The highest humoral immune response was observed in the Swiss volunteers, especially following the third dose immunized with 50 μg of candidate vaccine formulated in GLA-SE, giving a median of 51,200 Ab titers (day 84); however, the titers decreased 26 weeks after the last immunization (9,600 median titer). Even though the anti-P27A Ab induced in the formulation with both adjuvants had less than 25% recognition of Tex, cell inhibition was only 10% in both cases. Nevertheless, the researchers explained that the Tanzanian volunteers’ reduced immune response was related to other types of infection, genetic factors (HLA) or previous exposure to malaria and stated that a higher dose of adjuvant was required for carrying out a new clinical trial (Steiner-Monard et al., 2018).

### BK-SE36 Vaccine

*Plasmodium falciparum* serine repeat antigen 5 (SERA-5) is an abundant protein during the asexual blood stage (Horii et al., 2010), having limited antigenic polymorphism (Palacpac et al., 2011). It is stored in the parasitophorous vacuole (PV) and released in soluble form for mediating schizont, Mrz, and gamete rupture and release. It is cleaved into three fragments: a 47-kDa N-terminal domain, a 50^∗^kDa central domain and an 18 kDa C terminal, from which a 6-kDa fragment is derived (Li et al., 2002).

*In vitro* trials have shown that mouse Ab against the 47-kDa N-terminal domain (SE47′) inhibited parasite intra-erythrocyte proliferation (i.e., considered a strategic region for vaccine development (Fox et al., 1997). However, its hydrophobic nature hampers its purification, leading to a recombinant molecule called SE36 being designed as vaccine candidate from a synthetic gene encoding *P. falciparum* aa residues 17–382 (Honduras-1 strain) (Horii, 2008; Horii et al., 2010). SE36 was expressed in *Escherichia coli* and formulated with AH gel (AHG) (Tougan et al., 2018).

Clinical phases Ia and Ib follow-up results have been published (Palacpac et al., 2013; Horii, 2014; Yagi et al., 2016) The first evaluated the vaccine’s safety and immunogenicity in 40 healthy, malaria-naive, Japanese male adults when administered by subcutaneous route in three doses on days 0, 21, and 42, at two different concentrations (50 and 100 μg of BK-SE36). Follow-up lasted 63 days, giving a good safety profile, high Ab response, and 100% seroconversion rate following the second dose, thereby enabling advancing to new clinical trial phases (Horii et al., 2010).

Phase Ib was developed in two stages in Uganda; the first involved healthy 21- to 40-year-old adults (*n* = 56) who were serologically negative or positive to anti-SE36 Ab. The second stage included healthy children and young adults (*n* = 84) in three age cohorts (16–20, 11–15, and 6–10 years). The most frequently occurring AE was induration (92% of participants in stage 1 and 63% in stage 2); there were no serious AE, meaning that the vaccine was considered safe. Regarding immunogenicity results, the change in Ab titers was only significant for 6- to 10-year-old participants (5.71-fold increase from baseline) vaccinated with 1.0 (100 μg) BK-SE36. The immunogenicity response with 0.5 (50 μg) BK-SE36 was low and not statistically significant (1.55-fold increase). It was concluded that the absence of a robust humoral immune response seemed to be common in malaria-endemic areas due to previous exposure to natural *P. falciparum* infection.

Complementary analysis with this study population demonstrated 21% (7/33 participants) malarial incidence in the group vaccinated with 1.0 BK-SE36, 30% (10/33) in the 0.5 BK-SE36 group, and 45% in a placebo group, following 1 year’s follow-up (Palacpac et al., 2013).

Based on these results, the relationship between human leukocyte antigen (HLA-DRβ1) allele polymorphism and host genetic factors was studied regarding developing an effective immune response to vaccination. Although previous research has demonstrated that HLA-DRβ1 influenced an immunogenic response against malaria [i.e., SPf66 (Murillo et al., 1991) and in the AMA-1 and rhoptry-associated protein (RAP2) vaccine candidates], this study determined that DRβ1 alleles did not influence Ab response to BK-SE36 or vaccinated subjects’ susceptibility to clinical malaria (Tougan et al., 2016). The vaccine is now being evaluated in a phase Ib trial in children under 5 years old (Tougan et al., 2016; Yagi et al., 2016).

### AMA-1 DiCo Vaccine

The *Pf*AMA1-DiCo recombinant vaccine candidate is based on the AMA-1 protein concentrated at the Mrz apical pole which participates in their reorientation and RBC invasion (Remarque et al., 2008b; Kwenti et al., 2017). This protein is stored in the micronemes (Srinivasan et al., 2011); it is structurally formed by 614 aa, having a 83-kDa molecular weight (Patarroyo et al., 2017a). It is differentiated into two regions; the first has a 550-aa ectodomain divided into three domains (I, II, and III) located in the N-terminal extreme, and the second, a transmembrane, intracytoplasmic region located in the C-terminal extreme (Lim et al., 2014; Lumkul et al., 2018). AMA-1 is a highly polymorphic antigen (Ouattara et al., 2010); induced Ab response mainly targets the protein’s functionally irrelevant and variable portions, thus distracting a host’s immune response (Wright and Rayner, 2014; Belachew, 2018).

The vaccine candidate includes three 454-aa (aa 100–545) sequences (DiCo 1, 2, and 3), designed by aligning and analyzing 355-aa sequences from the PfAMA1 FVO, HB3, and 3D7 strains’ extracellular region, covering 97% of target aa variability. A synthetic gene was constructed for each of the three DiCo sequences and then expressed in the yeast *Pichia pastoris* (Remarque et al., 2008a). A stepped phase Ia/Ib AMA-1 DiCo clinical trial was carried out in Europe and Africa. Phase Ia in Paris included 30 healthy adults who received three intramuscular (IM) doses of 50 μg of AMA-1 DiCo on days 0, 28, and 182, with two different adjuvants (alhydrogel, *n* = 15, or GLA-SE, *n* = 15). Phase Ib in Burkina Faso included 36 participants having had previous exposure to malaria, who received 50 μg of AMA-1-DiCo with GLA-SE (*n* = 18) or placebo with saline solution (*n* = 18). The vaccine was well tolerated by all participants; the most frequently occurring local reaction was pain at the injection site. Both vaccine formulations were immunogenic; nevertheless, GLA-SE was more potent than alhydrogel in the French participants, having a 200–300-fold increase in IgG Ab titers. By comparison, most volunteers in Burkina Faso who were seropositive during screening and had a lower Ab titer increase (four times) had higher IgG concentrations than the European participants by the end of the study (Sirima et al., 2017).

Considering that previous studies’ testing vaccine candidates based on variable AMA-1 regions have shown high immunogenicity but poor protective efficacy (Sagara et al., 2009; Payne et al., 2016), it remains to be seen whether AMA-1-DiCo (which also includes polymorphic regions) can achieve higher protection-inducing efficacy in ongoing phase II trials.

### Placental Malaria Vaccines: PRIMVAC and PAMVAC

Pregnant women represent a group of the population having a greater risk of contracting malaria (Gbédandé et al., 2017). *P. falciparum* infection leading to the expression of the VAR2CSA antigen (a variable member of the PfEMP1 protein family interacting with chondroitin sulfate A) facilitates the accumulation of infected erythrocytes in the placenta, causing many obstetric complications, such as the threat of preterm delivery, low birth-weight, gestational anemia, and greater susceptibility to the parasite during infancy. This often leads to preterm mortality, involving around 100,000 abortions per year and thus making it a serious public health problem (Gbédandé et al., 2017).

Two anti-placental malaria vaccine candidates were presented at the European Vaccine Initiative (EVI) in Paris and Brussels in 2014; PRIMVAC and PAMVAC are based on the VAR2CSA protein. The first includes Duffy binding-like (DBL) regions 1X and 2X (DBL1X-DBL2X) and a 105-kDa VAR2CSA domain (Figure 2E) from the *P. falciparum* 3D7 strain, all expressed as a recombinant protein in *E. coli* (Juillerat et al., 2011). A preclinical study involved BALB/c mice that received four doses IM of 110 μg PRIMVAC formulated with two adjuvants (GLA-SE or alhydrogel) on days 1, 15, 29, and 43. The pharmacological product was stable and remained potent up to 3 years after having been stored at −20°C, and no toxicity was observed. PRIMVAC adjuvanted with alhydrogel or GLA-SE produced Abs which could recognize VAR2CSA expressed on the surface of erythrocytes infected by different strains. The mice immunized with either of the two adjuvants had high Ab values (≥1/819,200) by day 65. These Abs also inhibited interaction with the homologous NF54–CSA strain and heterologous strains with CSA to a lesser extent (Chêne et al., 2019).

Clinical phase Ia/Ib began in January 2016; it involved 68 healthy 18- to 35-year-old European and African adults who received three doses (20, 50, and 100 μg) of PRIMVAC by IM injection on days 0, 28, and 56. It was estimated that this first clinical trial would culminate in April 2018; however, no preliminary research results are available (Srivastava et al., 2010; Dechavanne et al., 2015; Sirima et al., 2016a).

PAMVAC is based on DBL inter-domains 1 and 2a (ID1-DBL2X-ID2a) as a 73-kDa fragment derived from the *P. falciparum* FCR3 strain *Pf*EMP1 protein (Figure 2E), produced as a recombinant protein in *Drosophila melanogaster* Schneider-2 (S2) cells. The Ia/Ib clinical phase with PAMVAC began in May 2016, involving 66 healthy adults from Germany and Benin who received the same vaccination scheme as used with PRIMVAC. Preliminary safety and immunogenicity results for this vaccine candidate remain unknown (Mordmüller and Issifou, 2018).

### PfPEBS Vaccine

**FIGURE 3:**
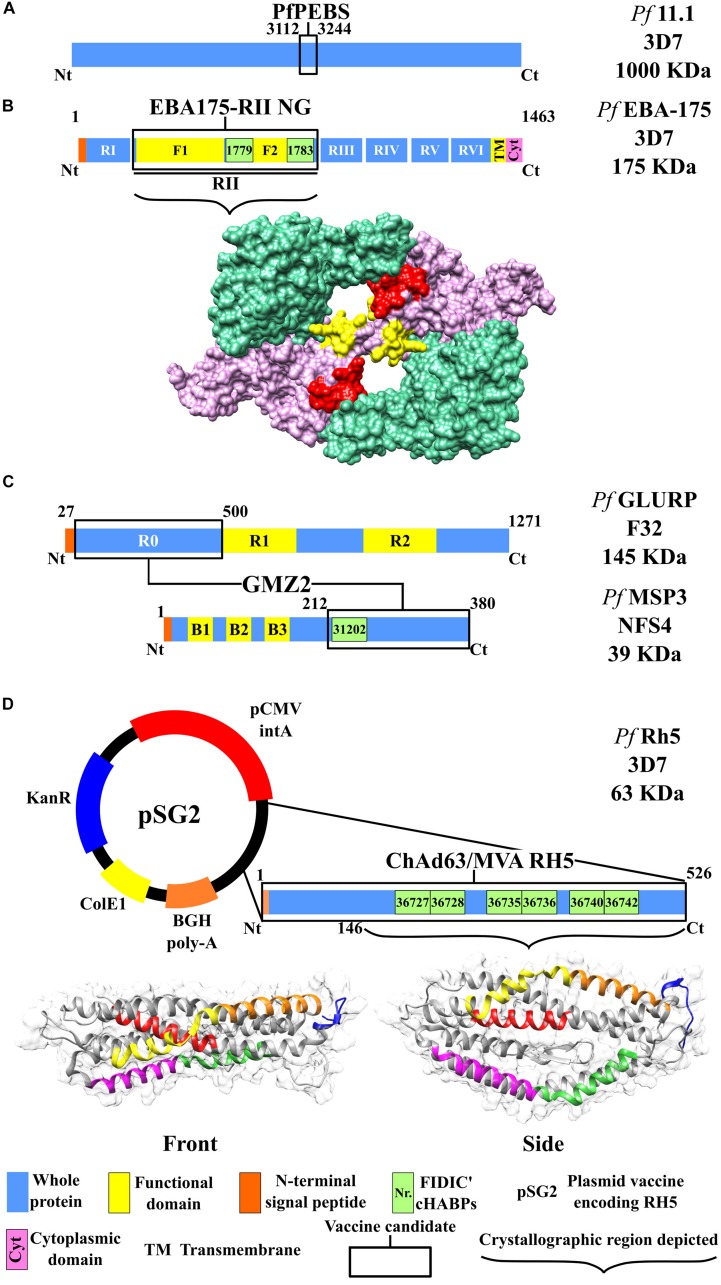
Scheme for the proteins used as candidates for an antimalarial vaccine (II). Color code according to that used in Figure 2. **(A)** PfPEBS vaccine. **(B)** Top: Scheme for the EBA-175 RII NG vaccine. Bottom: Protein structure, including region II surface, F1 (green) and F2 domains (pink), and conserved high-activity binding peptides (cHABPs) 1779 (red) and 1783 (yellow), PDB 1ZRO (Tolia et al., 2005). **(C)** GMZ2 vaccine, including the scheme for proteins MSP3 and GLURP according to the color code used for Figure 2. **(D)** ChAd63/MVA RH5 vaccine. Top: pSG2 plasmid, including the kanamycin resistance gene (*KanR*), cytomegalovirus (CMV) with the associated intron A (CMVintA), bovine growth hormone (bGH) with polyadenylation (bGH PolyA), *Escherichia coli* β-galactosidase (ColE1), and the RH5-encoding gene. Bottom: Surface and ribbon for the RH5 protein with cHABPs 36727 (magenta), 36728 (green), 36735 (yellow), 36736 (orange), 36740 (red), and 36742 (blue). PDB 4WAT (Chen et al., 2014).

This vaccine uses a 1,000-kDa fragment (Figure 3A) from the *P. falciparum* Pf11.1 antigen (Petersen et al., 1990) which is expressed during pre-erythrocyte and blood stages (*Pf*PEBS) which have been demonstrated to have *in vitro* functional activity against pre-erythrocyte and erythrocyte stages during *P. falciparum* infection. The vaccine is made as a synthetic protein and administered with AH adjuvant in two doses with a 28-day interval. It was postulated that 36 healthy 18- to 45-year-old adult volunteers should be included in phase Ia/II; they would have randomly received 5 or 30 μg of the vaccine for evaluating vaccine safety and immunogenicity profile as well as its efficacy through challenge studies. However, no data regarding this vaccine candidate’s clinical study’s preliminary results in humans have been published to date (Druilhe and Genton, 2012).

### EBA-175 RII NG Vaccine

The EBA-175 (175-kDa) protein belongs to the erythrocyte binding protein (EBP) family, which enables Mrz to interact with RBC and whose presence or absence defines alternative invasion routes (Zerka et al., 2016). The EBA-175 structure is formed by eight regions: a transmembrane one, a cytoplasmic one, and six extracellular regions or domains (I–VI). Domain II has been the most studied to date; it has functional importance regarding specific erythrocyte binding, to glycophorin A (GYPA). However, it has been found recently that domains V and VI, being translocated to the membrane, also mediate RBC invasion. The domain II region consists of 616 aa, having two cysteine-rich regions (F1 and F2) which are homologous for the *Plasmodium vivax* Duffy binding protein (Narum et al., 2000) (i.e., DBL for Duffy binding-like domain).

The vaccine developed with this non-glycosylated antigen (EBA-175 RII NG) contains F1 (aa 8–282) and F2 regions (aa 297–603) in its sequence (Tolia et al., 2005), thereby enabling binding to host RBC GYPA, and was expressed in *P. pastoris* (Zhang and Pan, 2005). *In vitro* studies have shown that malaria can be attenuated by the production of high Ab levels targeting EBA-175 (Narum et al., 2000; Ohas et al., 2004), making it a potential vaccine candidate.

A Ia phase clinical trial included 80 participants in the USA who received three doses of the vaccine on days 0, 42, and 194 at different concentrations (5, 20, 80, and 160 μg), having slight- to moderate-intensity local and systemic reactions. Immunogenicity results highlighted appreciable anti-EBA-175 Ab levels when greater than 20-μg concentrations were administered with at least two immunizations. All participants vaccinated developed *in vitro* parasite growth during inhibition trials (3D7 strain) 14 days after the third dose (day 194) but had insufficient ability for inhibiting *P. falciparum* growth, ranging from 7% to 19% (El Sahly et al., 2010).

An additional randomized, double-blind, phase Ib study evaluated the safety and immunogenicity of EBA-175 RII NG adjuvanted with aluminum phosphate administered in three doses to 60 semi-immune adults in Ghana (Africa) (Koram et al., 2016). Vaccine concentrations (5, 20, and 80 μg) were well tolerated; however, 38 individuals developed grade 1 and grade 2 adverse reactions during follow-up (no severe AE). Ab titer geometric mean was similar for all vaccinated groups, decreasing after day 42 and rapidly increasing after the third vaccination. The highest parasite growth inhibition percentage occurred in the placebo group (25.0%) compared to the vaccinated group (12.0–20.0%).

Some limitations found in both trials were related to sample size, short/limited follow-up, and differences between immunological response regarding vaccine concentrations between people exposed to malaria and those not exposed to it. More time must thus be dedicated to developing the EBA-175 RII NG vaccine to improve its Ab functionality due to poor *P. falciparum* growth inhibition as demonstrated in *in vitro* trials (Koram et al., 2016).

### GMZ2 Vaccine (a Recombinant Protein Consisting of GLURP and MSP3 Conserved Domains)

GMZ2 is a fusion vaccine which includes N-terminal domain non-repeat regions from soluble (released to the environment during parasite growth) and glutamate-rich GLURP (R0 N-term-GLURP_27__–__500_) proteins, identified as the main B-cell epitopes. It has a 220-kDa molecular weight and is expressed during *P. falciparum* pre-erythrocyte and erythrocyte stages (Singh et al., 2004). The vaccine also includes a 48-kDa molecular weight, MSP3 (MSP3_212__–__380_) C-terminal domain conserved region (Figure 3C) (Liang and Sim, 1997; Theisen et al., 2017). It has been proven that these antigens can induce Abs in semi-immune individuals (Meraldi et al., 2004; Soe et al., 2004).

A phase Ia clinical trial was carried out in Germany in 2006 involving 30 participants who were divided into three groups according to the dose being administered (10, 30, or 100 μg GMZ2) (Esen et al., 2009). The vaccine proved safe and was well tolerated; all adverse reactions were grade 1 or 2, being mainly associated with headache, fatigue, and nausea. Mean anti-GMZ2 Ab concentration similarly increased following the third vaccination, having an average of 1.20 mg/dl values in group 1, 1.60 mg/dl in group 2, and 1.33 mg/dl in group 3; however, such values considerably decreased following 1 year’s follow-up (day 365) regarding initial concentrations.

Phase Ib involved 40 participants in Gabon (Africa) who received three doses of 100 μg GMZ2 (Mordmüller et al., 2010). No severe AE (grade III) were reported, even though four participants developed malaria in the control group (anti-rabies vaccine) and in the group vaccinated with GMZ2. Although vaccinated individuals had higher anti-GMZ2 with 1.4 Ab levels higher than those for the control group at the end of the study, there was no difference regarding Ab production and memory B-cell response, possibly produced by intense previous exposure to *P. falciparum.*

Phase II clinical trials were carried out for evaluating GMZ2 efficacy. One of them involved thirty 1- to 5-year-old Gabonese children who randomly received three doses of 30 or 100 μg GMZ2 or anti-rabies vaccine (control group) on days 0, 28, and 56, with a 1-year follow-up (Bélard et al., 2011). Safety, reactogenicity, and immunogenicity results were similar to those for the study’s first phase, and the geometric mean for Abs produced was higher for the group which received the vaccine at 100-μg GMZ2 concentration (this being the dose selected for a multicenter trial).

This new study (phase IIb) in Burkina Faso, Ghana, Uganda, and Gabon involved immunizing 1,849 1- to 5-year-old African children; 868 of them received three doses of 100 μg GMZ2 at 0-, 1-, and 6-month intervals; 867 received the anti-rabies vaccine (control); and 114 were withdrawn from the trial (Sirima et al., 2016b). The GMZ2S group suffered 641 episodes of malaria and 720 occurred in the control group; 32 cases were severe malaria. Vaccine efficacy was 14% (adjusted for age and site) and 11.3% (adjusted for age), indicating that GMZ2 formulation must be improved to enable clinical studies to continue. Greater, longer-lasting immune response is thus required for inducing significant protection against clinical disease which could stimulate memory B-cells and long-lived plasma cells (LLPC) (Theisen et al., 2017).

### ChAd 63RH5/MVA RH5 Vaccine

The reticulocyte binding protein homolog 5 (PfRH5) has a 63-kDa molecular weight (526 aa), this being lower than other RH family proteins (RH1, RH2a, RH2b, and RH4) ranging from 200 to 375 kDa (Arévalo-Pinzón et al., 2012; Chen et al., 2014). RH5 is a necessary ligand for *P. falciparum* invasion of erythrocytes and plays an essential role in parasite survival via its interaction with RBC surface receptor basigin (BSG) (Baum et al., 2009). Furthermore, it must be associated with cysteine-rich protective antigen (CyRPA) and PfRh5-interacting protein (PfRipr) to completely carry out its function, forming a macromolecular complex mediating RBC entry (Reddy et al., 2015; Wong et al., 2019). RH5 adopts different conformational states during complex formation, particularly when interacting with CyRPA and Ripr, such molecular rearrangements being crucial for target cell entry.

Structural analysis of RH protein family erythrocyte binding has led to identifying new inhibiting epitopes (Wright et al., 2014). Studies concerning anti-*Pf*RH5 monoclonal Ab (mAb) have indicated complete inhibition against parasite invasion of RBC *in vitro* (Ord et al., 2014); this has been associated with protection against the disease (Ord et al., 2015). ChAd63 and MVA were used for developing the vaccine (Payne et al., 2017) (Figure 3D).

A phase Ia clinical trial for comparing/evaluating ChAd63 RH5 cf ChAd63 RH5 + MVA was carried out at two concentrations; it involved 24 British adults who had not been exposed to malaria (Payne et al., 2017). The vaccine had a favorable safety profile; most slight or moderate AE occurred in the MVA groups. The ChAd63 and MVA vectors were designed to maximize Ab response induction against erythrocyte-stage malaria antigens; reactogenicity, kinetics, and the magnitude of CD4+/CD8+ T-cell response results agreed with those found in other vaccine trials carried out with similar doses of these vectors (Sheehy et al., 2011, 2012). This was consistent with the hypothesis that immunity is mainly associated with the viral vector.

*In vitro* parasite growth inhibition trials involving a growth inhibition assay (GIA) gave 36.0–50.6% using 10 mg/ml purified IgG of immunized individuals. Avidity-based extracellular protein interaction screening (AVEXIS) tests confirmed that the vaccine could also block RH5FL complex proteins’ interaction with P113, CyRPA, and BSG proteins (Payne et al., 2017), forming a trimolecular complex for mediating RBC invasion. Subsequent studies analyzed the kinetics of Ab responses targeting RH5 complex antigens (PfRH5, CyRPA, and Pf113) in Ghanaian children suffering from *P. falciparum* malaria (Partey et al., 2018). The results showed that IgG-specific Abs became induced against PfRH5 complex proteins during acute malaria but that their prevalence was low and IgG levels rapidly decreased following treatment. Such data indicated that PfRH5 complex-specific Ab levels in natural infection in Ghanaian children were only markers for recent exposure (Partey et al., 2018).

Abs induced by immunization with ChAd 63RH5/MVA RH5 might not fully block RH5–BSG interaction since it has been shown that RH5 interacts better with RBC when complexed with Ripr and CyRPA than when binding alone (Wong et al., 2019). Moreover, it has been shown that the inhibitory effect of sera raised against RH5 and sera raised against CyRPA is additive (Favuzza et al., 2017); including both antigens in a vaccine formulation would thus be desirable. It remains to be seen whether protective efficacy increases when all the proteins belonging to the complex are formulated together as RH5 conformation shifts when alone or in complex (Chen et al., 2014) and whether RH5–CyRPA–Ripr complex formation is essential for target cell entry.

## Concluding Remarks

Developing vaccines against *P. falciparum* erythrocyte-stage infection has mainly been focused on formulations involving single-antigen recombinant proteins from a limited amount of molecules or their fragments (two or three). Most have had an acceptable safety profile and have sometimes been able to induce low or medium Ab titers partially capable of inhibiting parasite growth *in vitro* (i.e., in a GIA). However, the immunogenicity so induced has not been sufficient for providing significant protection against malaria, given the parasite’s tremendous complexity (which involves many proteins and mechanisms during invasion), the parasite’s genotype and phenotypical diversity (for evading a host’s immune system), the receptor molecules’ genetic variability, and human immune system molecules’ tremendous genetic complexity.

Developing an effective vaccine thus implies a clear understanding of the parasite’s life cycle, its antigenic polymorphism, sialic acid-dependent and sialic acid-independent invasion routes or alternative pathways, and a suitable production system regarding complete proteins or their functionally active basic subunits. An adjuvant or vaccine delivery system must be selected which can boost the immune response and guarantee a suitable safety profile.

The main difficulty regarding currently available vaccines (in trials) is that a high Ab concentration must be induced for blocking erythrocyte invasion and that they involve using antigens inducing specific immunogenicity against the vaccine strain which does not correlate with a person’s real exposure to different parasite strains in natural settings. The methods used for this type of formulation today involve using naked DNA encoding specific proteins, complete recombinant proteins, or viral vector DNA, some of which are associated with developing high rates of local and systemic reactogenicity.

No vaccine encoding a single blood-stage antigen or protein has induced significant immunogenicity and/or protection in phase I/II trials to date; new clinical studies should thus include more participants having different age ranges, thereby enabling statistically significant analysis and prolonged follow-up time for evaluating immunological memory. The formulation of a vaccine which can induce both humoral and cell-mediated immune responses would enable inducing immunity against *P. falciparum*, thereby increasing the/any efficacy reported to date.

A new functional approach recently suggested by other groups is that a fully protective antimalarial vaccine must be multi-epitope and multistage, an approach which has long been claimed by us. This is due to multiple strategies, like redundant proteins performing similar or the same biological function, rapid mutation, in one or several aa to bypass the immune system with one single aa change. It also must be multistage, that is, targeting both pre-erythrocyte (sporozoite) and erythrocyte (Mrz) stages (different proteins according to proteomics) and involving alternative pathways due to different receptors on invaded cells or a strong immune response against targeted molecules to ensure that hepatocyte and RBC invasion can be avoided. It must also have transmission blocking (gametocytes) components to avoid the parasite cycle becoming completed to control or reduce transmission from humans to mosquitoes.

### Our Approach

A new functional approach to vaccine production is thus required when one considers the problems encountered regarding current vaccine candidates. FIDIC’s experience in the field (ca. 35 years) led to postulating a scientific, logical, and rational approach. This states that a fully protective antimalarial vaccine must be multi-epitope, multistage, and minimal subunit based, containing conserved aa sequences from key proteins by identifying high-activity binding peptides (cHABP); this can only be achieved by chemical synthesis.

Some of the many cHABPs identified by our institute have been included in the protein fragments selected by other groups for their vaccine candidate production. MSP3-derived cHABP 31202 (^201^Y^220^I) has been located in the MSP3-LSP vaccine’s conserved 95-aa fragment (Figure 2A). SERA-5-derived cHABPs 6725 (^141^Y–^160^K) and 6733 (^321^Y–^340^S) (Patarroyo et al., 2011; Bermúdez et al., 2012) are included in the vaccine candidate BK-SE36 recombinant fragment (Figure 2C). P*f*AMA1-derived cHABP 4313 (^134^D–^153^G) located in conserved domain 1 and cHABP 4325 (^374^M–^393^H) in domain 2 are included in the AMA-1 DiCo vaccine candidate (Figure 2D). The FIDIC group has described cHABPs 1779 (^356^N–^375^I) and 1783 (^436^H–^455^K) in the F2 region in the aa sequence used in the EBA-175 RII NG vaccine (Figure 3B) (Patarroyo et al., 2017a).

The blood-stage antimalarial vaccine candidates’ immunogenicity results described here (mainly based on *P. falciparum* protein conserved regions) have indicated that although moderate Ab titers are induced after a first immunization, they do not induce long-lasting protective immunity. This is consistent with our research demonstrating that, although conserved regions are functionally important for target cell binding, they are poorly recognized by host immune system due to being located far from the highly polymorphic regions used by the parasite as an evasion mechanism to distract the immune system (Patarroyo et al., 2017b); such polymorphic regions are immunodominant but confer just strain-specific immunity (Hisaeda et al., 2005; Curtidor et al., 2017).

cHABPs are immunologically silent due to the particular and specific 3D structure they adopt (Patarroyo et al., 2016, 2017a), thereby hampering an appropriate presentation to the major histocompatibility complex class II (MHC II) binding groove (an essential step for mounting a strong adaptive immune response). A further refinement requires that cHABPs must be appropriately modified (mHABPs) to cover the immune system’s (HLA) immense human genetic variability, thus named immune protection-inducing protein structures (IMPiPS). mHABPs have proven highly immunogenic and protection inducing against experimental malaria in *Aotus* monkeys. This experimental model is susceptible to human malaria, having an immune system 90–100% identical to humans, as demonstrated by DNA sequencing (Suárez et al., 2006), thereby explaining the aforementioned proteins’ suitable profiles as vaccine candidates (Patarroyo et al., 2011, 2017b; Rodriguez et al., 2008).

Animal trials involved immunizing from five to nine *Aotus* monkeys per group with 125 μg each of mHABP (Patarroyo et al., 2017a). Blood samples were obtained before each immunization (days 0, 20, and 40) and 20 days after the third dose for immunological studies. Immunized and control *Aotus* were intravenously injected with 100,000 *P. falciparum* FVO-strain-infected RBC to evaluate protective efficacy (defined as the total absence of parasites in blood during the 15 days of the test) (Rodriguez et al., 1990). Animals were treated with antimalarial drugs and kept in quarantine until ascertaining that they were parasite free and then returned to the jungle 4 weeks later according to National Institute of Health Animal Management Guidelines.

This methodology has enabled testing and selecting mHABP derived from parasite life cycle stages, which has led to promising results in obtaining a highly immunogenic and protection-inducing antimalarial vaccine (Garcia et al., 2007; Rodriguez et al., 2008; Bermúdez et al., 2012; Curtidor et al., 2017). Chemically synthesized vaccines’ main advantages include their high yield, reproducibility, purity (free of contaminants, such as endotoxins), lack of secondary adverse reactions, being able to synthesize unlimited amounts and stability at room temperature for long periods of time (not requiring cold chain) (Bermúdez et al., 2008; Skwarczynski and Toth, 2016).

The clear relationship observed between mHABPs’ structure, their immunological properties, and feasible production highlights the challenges and opportunities arising from this new methodology, along with the universal principles and rules used in developing a new vaccine.

## Author Contributions

DS, MG, LC-C, AC, and JM-F conceived the work and drafted the manuscript. CR designed the figures. MEP and MAP conceived the general work and critically revised the manuscript. All authors have revised the manuscript and given their approval for this version to be submitted.

## Conflict of Interest

The authors declare that the research was conducted in the absence of any commercial or financial relationships that could be construed as a potential conflict of interest.
